# Supplementary material for: Spatial transcriptomics identifies molecular niche dysregulation associated with distal lung remodeling in pulmonary fibrosis
Source: Nat Genet. 2025 Feb 3;57(3):647–58. doi: 10.1038/s41588-025-02080-x (PMC11906353; doi:10.1038/s41588-025-02080-x)
Supplement: Supplementary file 1 — Supplementary Figs. 1–22 and Supplementary Note. [file 41588_2025_2080_MOESM1_ESM.pdf]

nature genetics

Supplementary information https://doi.org/10.1038/s41588-025-02080-x

Spatial transcriptomics identifies molecular niche dysregulation associated with distal lung remodeling in pulmonary fibrosis

In the format provided by the authors and unedited

Supplementary Note

Cellular and molecular characterization of disease pathology

In one analysis, we compared cell-type composition across the unaffected, less affected, and more affected categories using *propeller*[36], which revealed significant differences in cell-type composition across groups for 28 cell-types (FDR < 0.05) (**Supplementary Fig. 5,6** and **Supplementary Table 4,5**). Compared to unaffected samples, disease groups had lower proportions of many cell-types typically associated with healthy alveoli, including AT1 and AT2 cells, capillary cells, and alveolar macrophages. In contrast, less and more affected samples had increased proportions of activated fibrotic fibroblasts, dendritic cell-types, B and T-cells, and venous cells, as well as *KRT5-/KRT17+*, basal, and respiratory airway secretory cells (RASCs). Overall, these results reveal dramatic changes in cellular composition of the PF lung, corroborating and refining insights from prior studies characterizing the cellular makeup and pathologic diversity of PF[6,17–23,37–42].

In addition to comparing across categories in this manner, the spatial heterogeneity of disease pathology in PF presents the opportunity to move beyond categorical designations of disease severity to capture the full spectrum of molecular and cellular dysregulation across subjects and samples. To this end, we calculated proportions of tissue exhibiting architectural changes (percent pathology) (**Extended Data Fig. 7a, Supplementary Table 6**) and tested for associations between changes in percent pathology and both cell-type composition and gene expression using linear regressions. We found that 24 cell-types and 154 genes (using subject-level pseudobulking) were significantly associated with percent pathology (FDR < 0.01) (**Extended Data Fig. 7b, Supplementary Fig. 7,8**, and **Supplementary Tables 7,8**). Generally, proportions of capillary and alveolar cells were associated negatively with percent pathology, as were many of their corresponding marker genes. The overall abundance of endothelial cells progressively decreased, but those remaining were more frequently venous-type in advanced pathology. By contrast, higher percent pathology scores were associated with an increased proportion of activated fibrotic fibroblasts and most lymphoid cell-types. Among myeloid cells, there was variability; broadly, the abundance of homeostatic-type alveolar and interstitial macrophages decreased, while dendritic cells became more frequent. We also performed a cell-type-aware analysis, identifying hundreds of genes that were significantly associated with percent pathology in individual cell-types (**Supplementary Fig. 9** and **Supplementary Data 1**). While previously noted considerations related to “contamination” of transcripts from adjacent cells introduce some challenges in interpreting these results, several notable findings emerge. For example, activated fibrotic fibroblasts express lower levels of *PDGFRA* but higher levels of *POSTN* in more fibrotic samples; subpleural fibroblasts exhibit higher levels of “inflammatory fibroblast markers” *CCL2* and *PTGDS. SPP1+* macrophages express higher levels of *FN1* and *CD86*, while transitional AT2 cells express lower levels of *RTKN2* and *LAMP3*, but higher levels of highly validated IPF biomarker *MMP7* and *KRT15.* Together, these results support not only large-scale compositional changes in advanced disease, but substantial changes in cell-type specific programs in pathologic conditions.

Next we sought to characterize the molecular and cellular basis of histopathologic features by annotating representative examples of 27 distinct features across samples (**Extended Data Fig. 7a,c**, and **Supplementary Fig. 10,11**; see **Methods**), including classical IPF-associated features (e.g., fibroblastic foci, microscopic honeycombing), general pathologic features (e.g., muscularized arteries, granulomas, goblet cell metaplasia, tertiary lymphoid structures (TLSs)), and additional features (e.g., remodeled epithelium, severe fibrosis, mixed inflammation). Characterizing the cellular diversity within these features then allowed us to establish key molecular processes underlying them (**Extended Data Fig. 7c** and **Supplementary Fig. 12,13**). For example, we observed that regions of granulomatous inflammation contained multiple macrophage subtypes in addition to T-cells. Regions of microscopic honeycombing were characterized by a low cuboidal epithelium expressing a variety of airway epithelial cell programs (including multiple secretory cell-types as well as basal cells), while regions of goblet cell metaplasia were located in larger airways and notable for the abundance of goblet cells and expression of *MUC5B* as the predominant airway mucin transcript. TLSs contained an expected T-cell predominance, while fibroblastic foci included not only activated fibrotic fibroblasts, but multiple fibroblast subtypes, smooth muscle cells (SMCs), and macrophages. Regions of hyperplastic alveolar epithelial remodeling were largely composed of “transitional AT2” cells co-expressing markers of AT1, AT2 and distal airway secretory cells.

Supporting Information

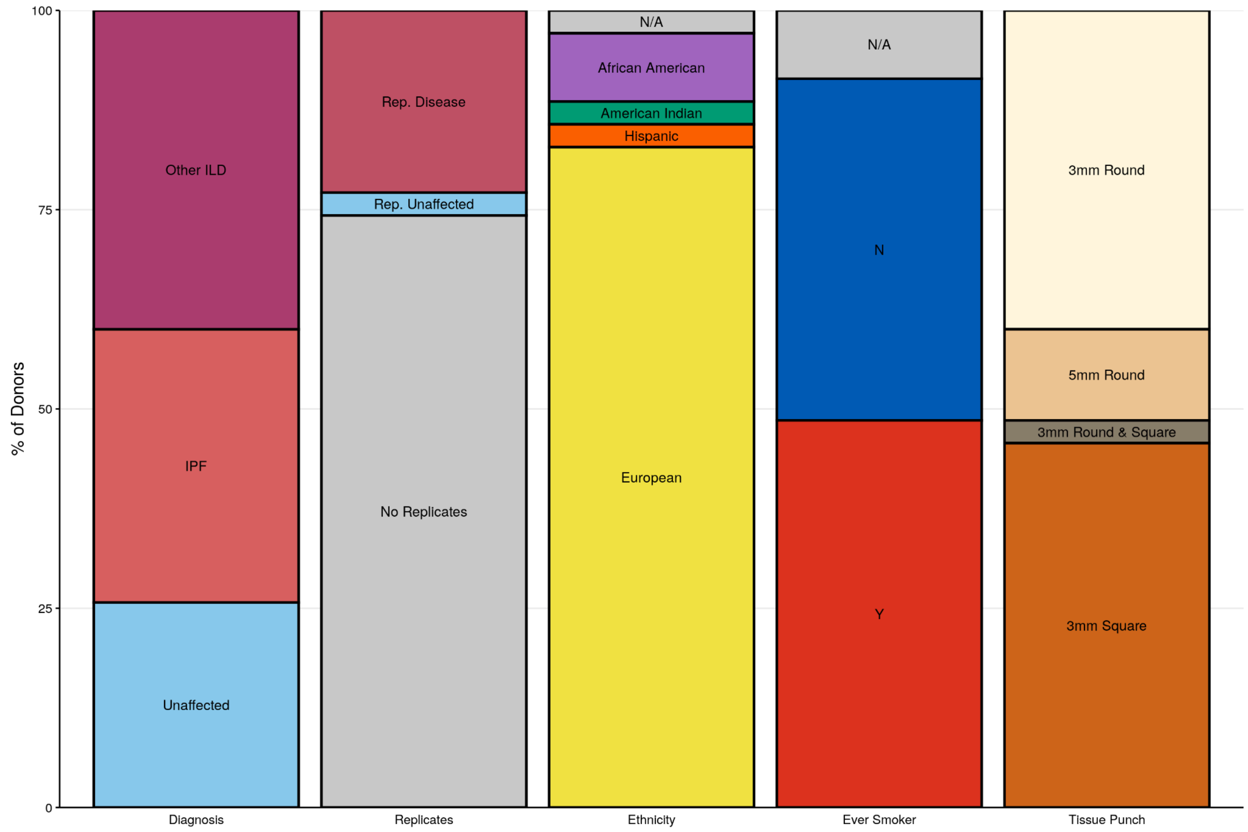

Supplementary Figure 1: Donor demographic information.
Samples (N = 45) were collected from 9 unaffected and 26 pulmonary fibrosis (PF) donors across several interstitial lung diseases (ILDs). The plurality of PF donors were diagnosed with idiopathic pulmonary fibrosis (IPF). For some donors, we collected 2 replicate samples for testing. Demographic information is also provided for reported ethnicity and smoking status. One donor (TILD117) had samples on TMAs with both 3mm round and 3mm square punches.

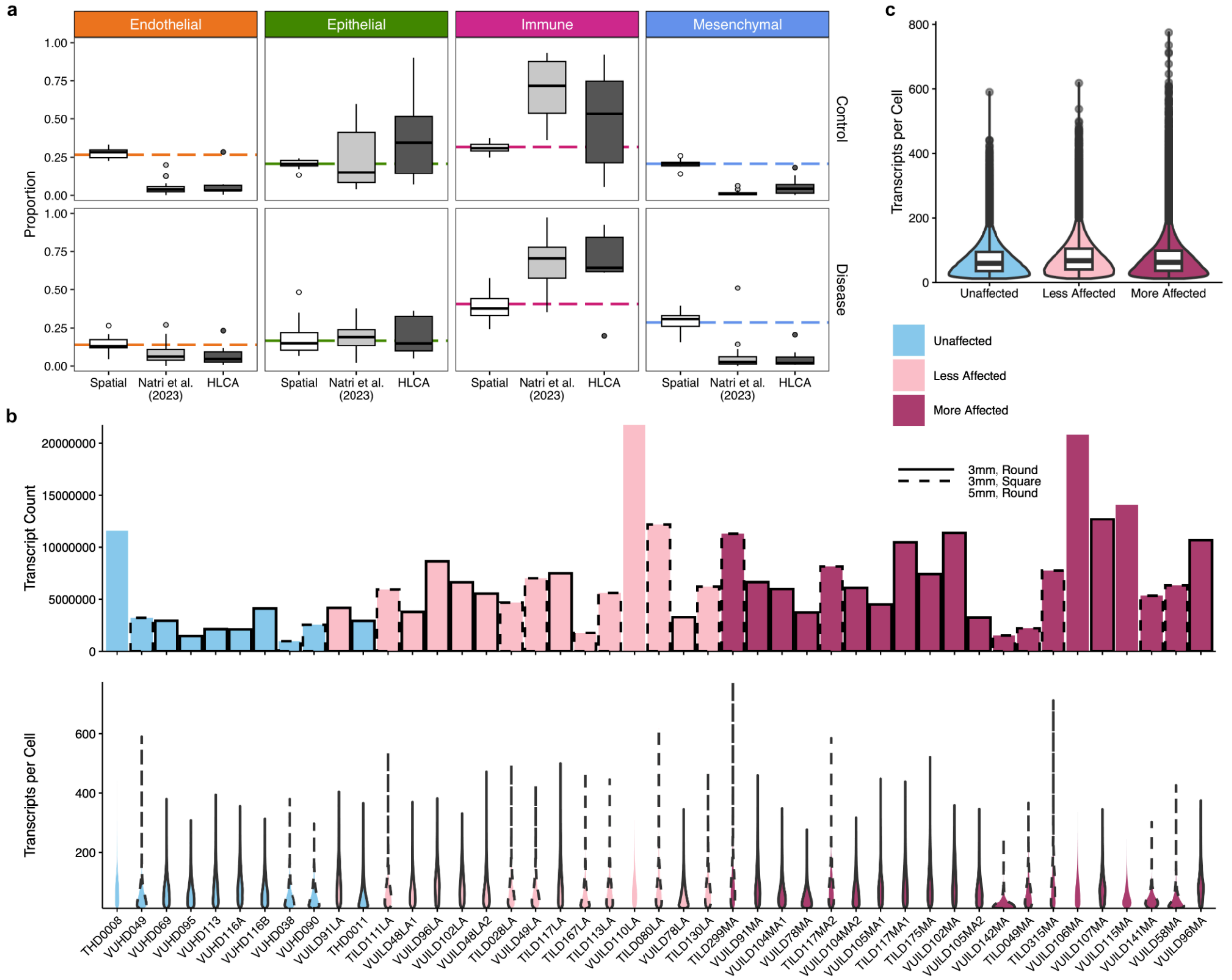

Supplementary Figure 2: Spatial transcriptomics detects a large number of transcripts across samples in unaffected and PF lungs.

a, Comparison of cell-type recovery between the present spatial dataset and prior single-cell RNA-sequencing (scRNA-seq) datasets from Natri et al. (2023)[1] and the Human Lung Cell Atlas (HLCA)[2]. Proportion of cells from each lineage was calculated per sample for the spatial dataset (10 control and 35 disease samples) and Natri et al. (2023; 19 control and 28 disease samples) and per dataset for HLCA (11 control and 7 disease datasets). For HLCA calculations, only studies that did not enrich or deplete for any cell lineage were included in the plot. Boxplots show the median, with the box hinges extending to the first and third quartiles and the whiskers extending to the largest (upper whisker) or smallest (lower whisker) value with a maximum of 1.5x the interquartile range above and below. Outliers are shown as individual points. Dashed lines indicate the overall proportion of cells in each lineage for the spatial dataset. **b**, Number of transcripts detected in each sample after filtering. Top: Bar plot of overall number of transcripts utilized in the GraphSAGE niche analysis (top), including transcripts not partitioned into a segmented cell/nucleus. Bottom: Transcripts per cell as a violin plot, only including transcripts that fell within segmented nuclei. Samples are arranged in increasing order of percent pathology (see Methods). Samples on TMA3 were larger than the other samples (5mm, round punches vs. 3mm square or round punches; denoted by no border, dashed, and solid lines, respectively; see

Methods) and had more detected transcripts. **c**, Number of transcripts per cell as in **b** (bottom), but split by sample group. Outlier points on the boxplots represent individual cells. Sample sizes are as follows: unaffected - 225,137 cells; less affected - 531,567 cells; more affected - 873,615 cells.

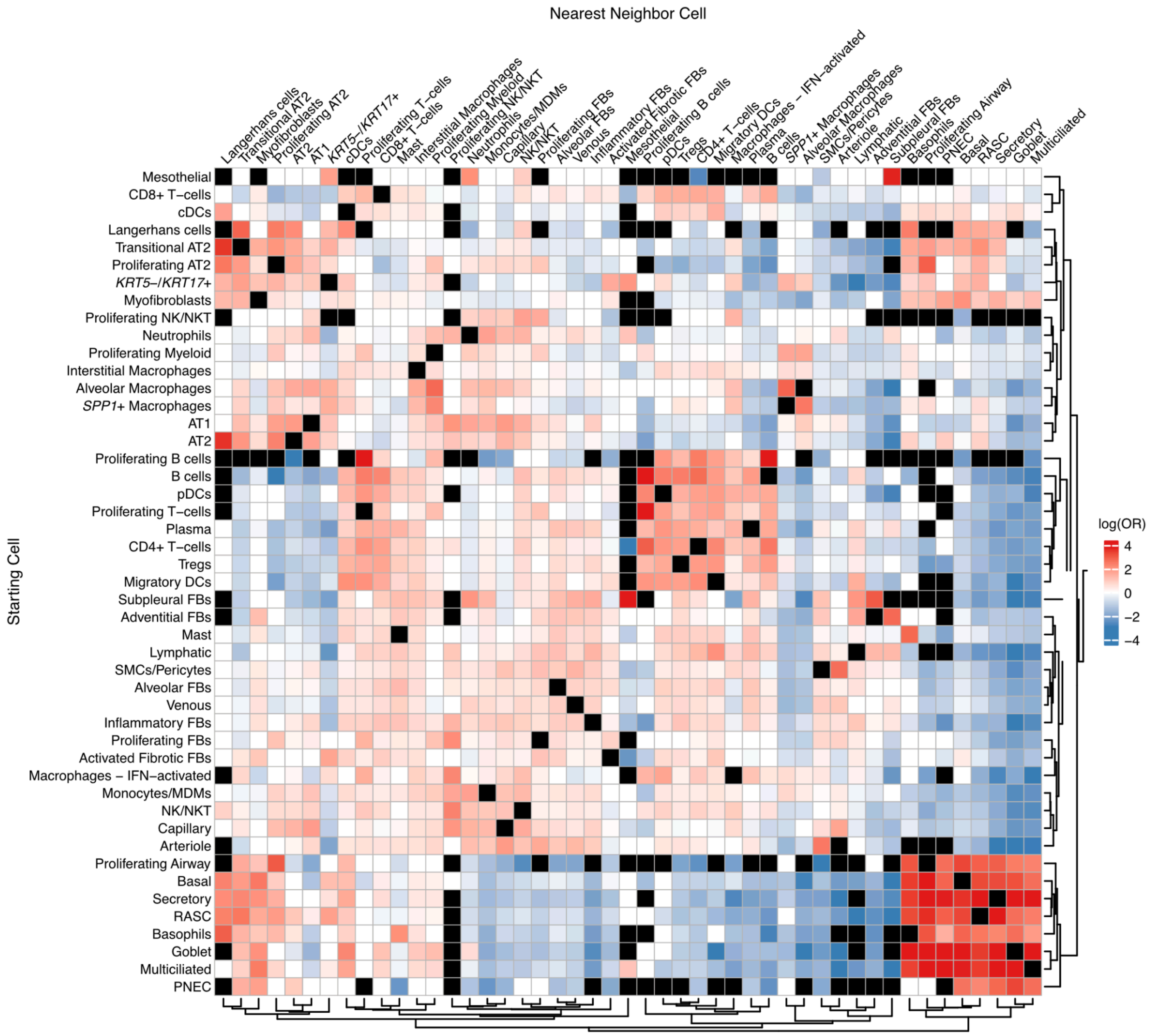

Supplementary Figure 3: Cell-type proximity results across all samples.
Heatmap showing odds ratio indicative of cell-type proximity likelihood relative to all other cells by logistic regression. Positive values represent cell-types that are proximal while negative values represent overall depletion. Black squares represent NAs where no cell was considered proximal between any cell-types pair.

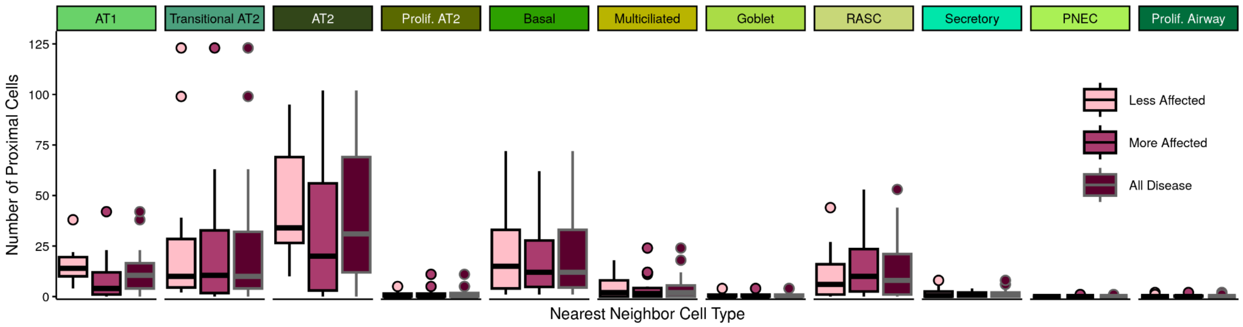

Supplementary Figure 4: Absolute proximity of *KRT5-/KRT17+* cells to other epithelial cell-types.

Boxplots show the total number of cells of each epithelial cell-type, excluding *KRT5-/KRT17+* cells, that were found to be the nearest neighboring cell to *KRT5-/KRT17+* cells in each sample. Boxplots show the distribution of total neighbor counts per sample (e.g., each point is a sample). The line in each box depicts the median, the box hinges extend to the first and third quartiles and the whiskers extend to the largest (upper whisker) or smallest (lower whisker) value with a maximum of 1.5x the interquartile range above and below the hinges. Outliers are shown as individual points.Results are shown separately for less affected and more affected samples, as well as for all disease samples together. Most *KRT5-/KRT17+* cells in direct proximity to epithelial cells were nearest to AT2 cells, followed by Transitional AT2, Basal, RASC, and AT1 cells.

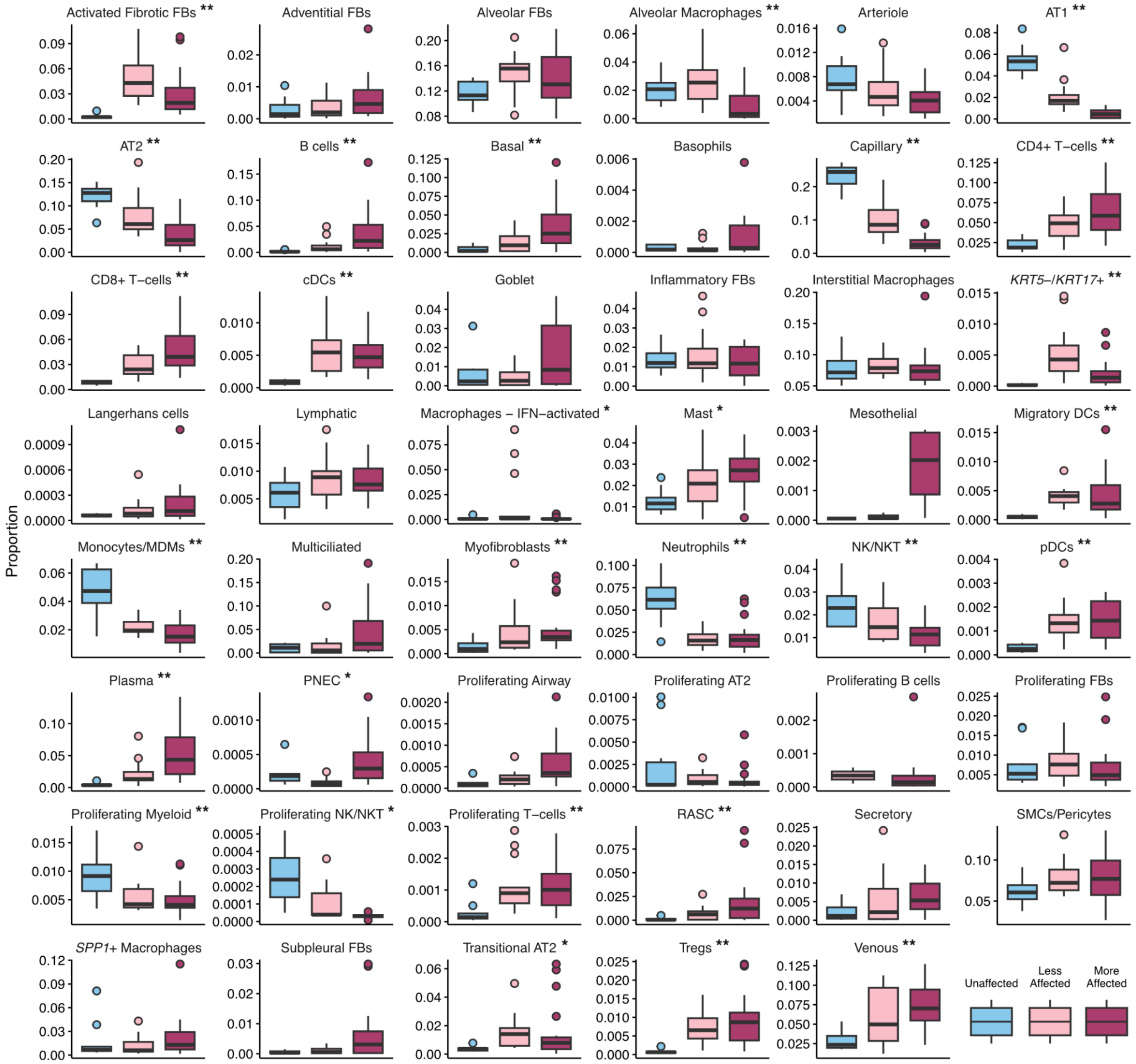

Supplementary Figure 5: Distribution of cell-types by disease severity.
Boxplots show the proportions of each cell-type across unaffected (n = 10), less affected (n = 15), and more affected (n = 20) samples. Boxplots show the median, with the box hinges extending to the first and third quartiles and the whiskers extending to the largest (upper whisker) or smallest (lower whisker) value with a maximum of 1.5x the interquartile range above and below. Outliers are shown as individual points. Proportions were logit-transformed for this analysis; here, raw proportions are shown for simplicity. ** and * indicate overall significant differences across the 3 groups (FDR < 0.01 and 0.05, respectively), via two-sided ANOVA tests

as implemented in *propeller*[3] (**Supplementary Table 4**). Post-hoc two-sided t-tests were also performed for significant ANOVAs (results reported in **Supplementary Table 5**).

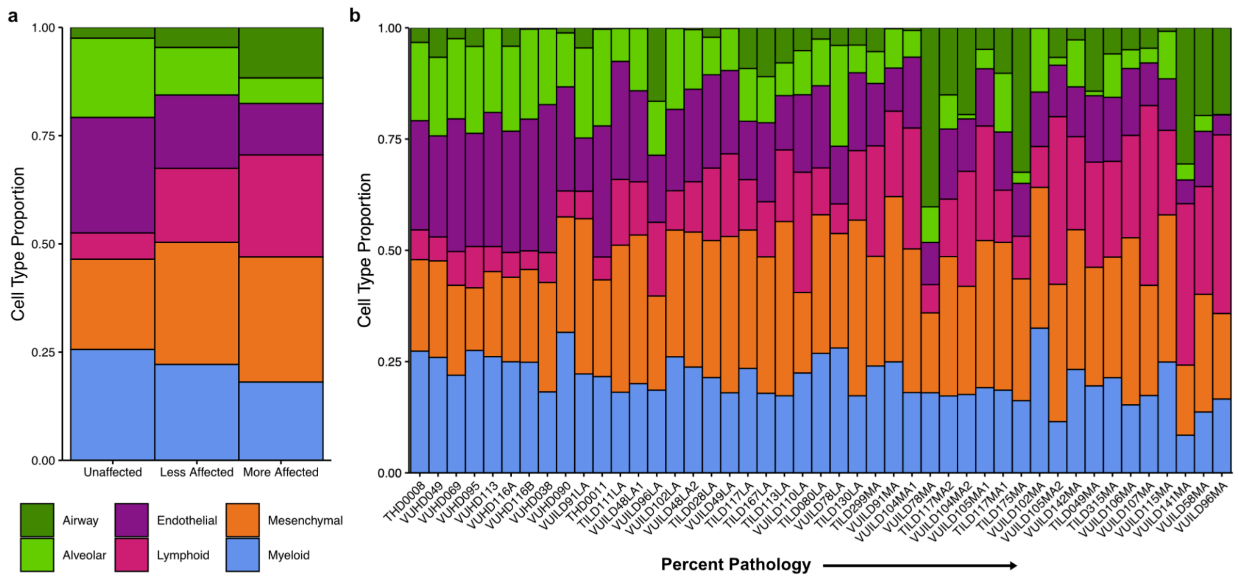

Supplementary Figure 6: Distribution of cell-types across samples.
Cell-type proportions across (**a**) sample type and (**b**) sample, ordered on the x axis by percent pathology from lowest to highest. Here, cell-types were sorted into broad categories for ease of visualization.

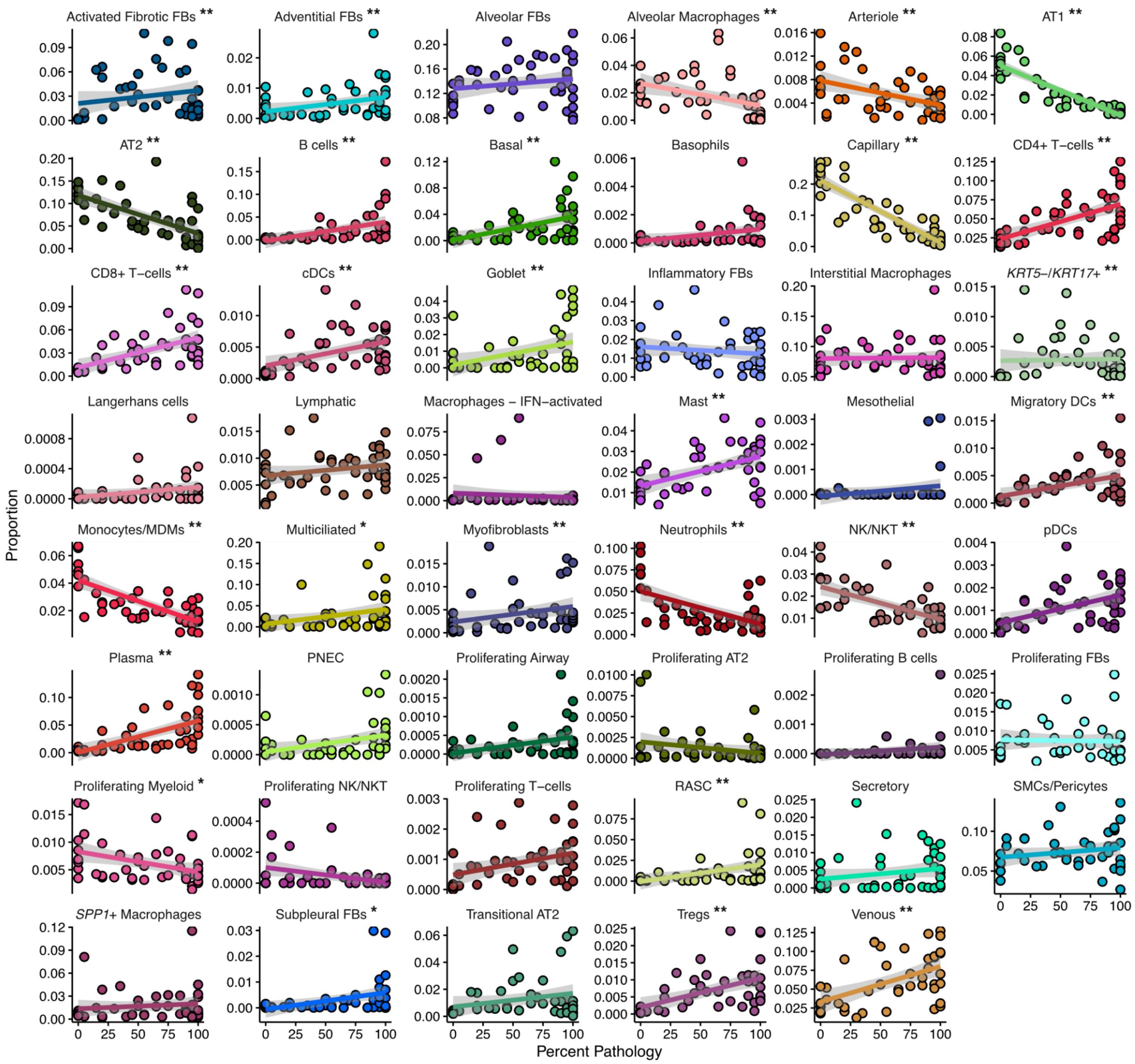

Supplementary Figure 7: Changes in cell-type composition with percent pathology. A linear model framework with *propeller*[3] and *limma*[4,5] was used to assess changes in cell-type proportions with changes in percent pathology across samples. Proportions were logit-transformed for this analysis; here, correlations of raw proportion with percent pathology are shown for simplicity. * and ** represent FDR < 0.01 and < 0.05, respectively, under the linear model (see Methods). Bands show 95% confidence intervals around the linear regression line.

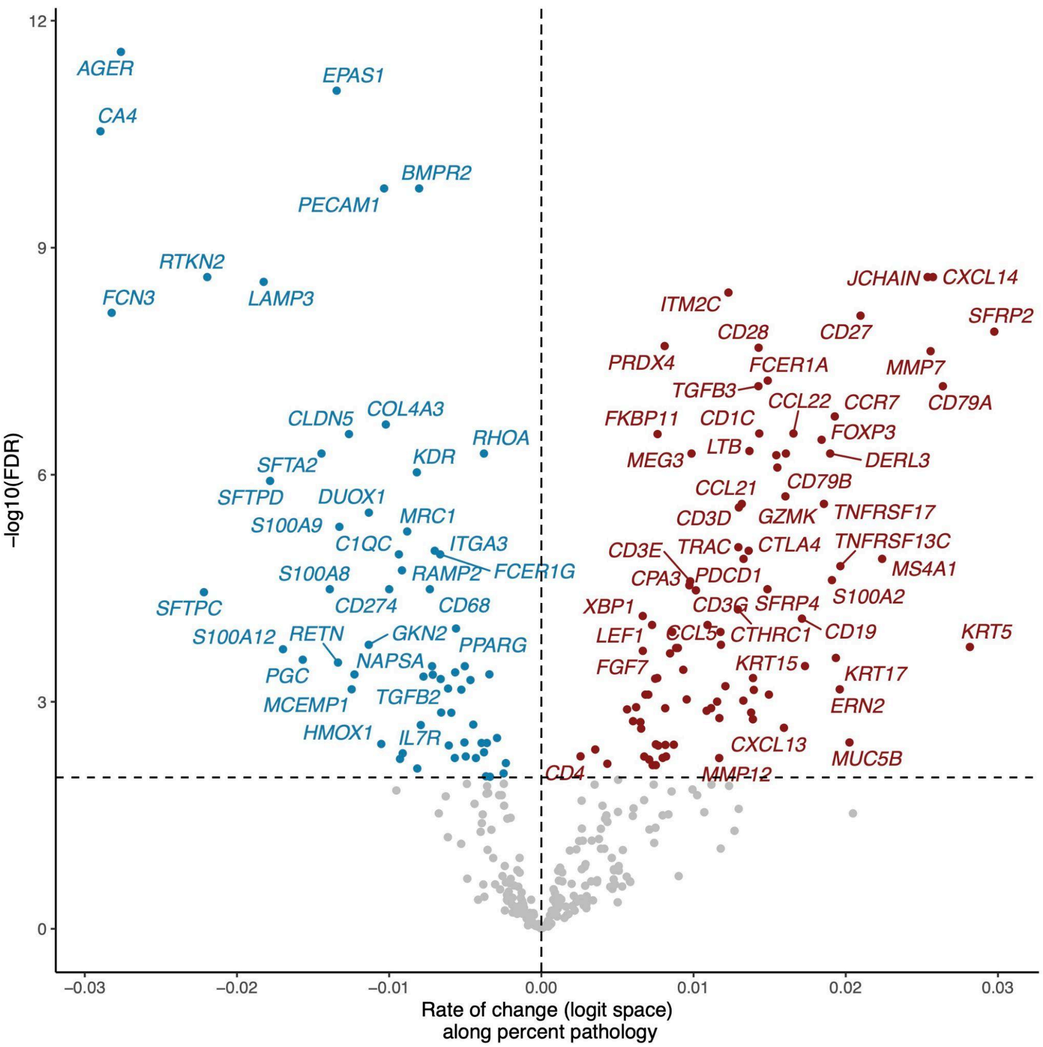

Supplementary Figure 8: Gene expression changes associated with percent pathology. Gene expression was aggregated per sample and converted to proportions. Linear models were fitted to each gene after logit transformation of gene proportions against percent pathology across samples. The horizontal and vertical dashed lines show the significance threshold (FDR < 0.01) and split the plot into genes negatively and positively associated with pathology score, respectively. Significant genes are colored and labeled.

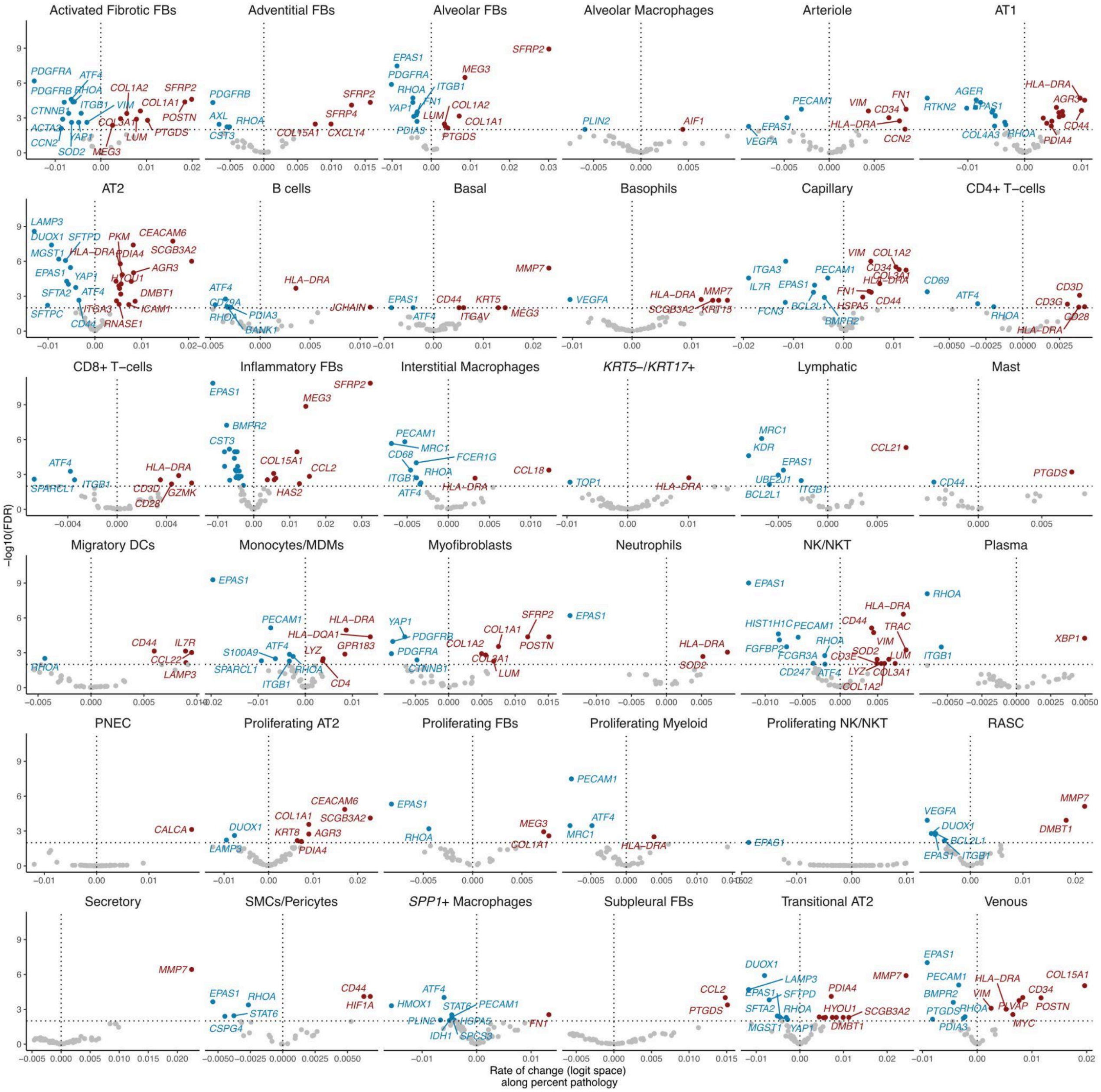

Supplementary Figure 9: cell-type-level gene expression changes associated with percent pathology.

Complementary analysis to **Supplementary Fig. 8**, identifying gene expression changes associated with pathology score across each cell-type. For each cell-type, we only tested genes that have been detected with >=5 copies in at least 30% of the testing cell-types after count-per-1k normalization across cells. The horizontal and vertical dashed lines show the significance threshold (FDR < 0.01) and split the plot into genes negatively and positively

associated with pathology score, respectively. Significant genes are colored and labeled.

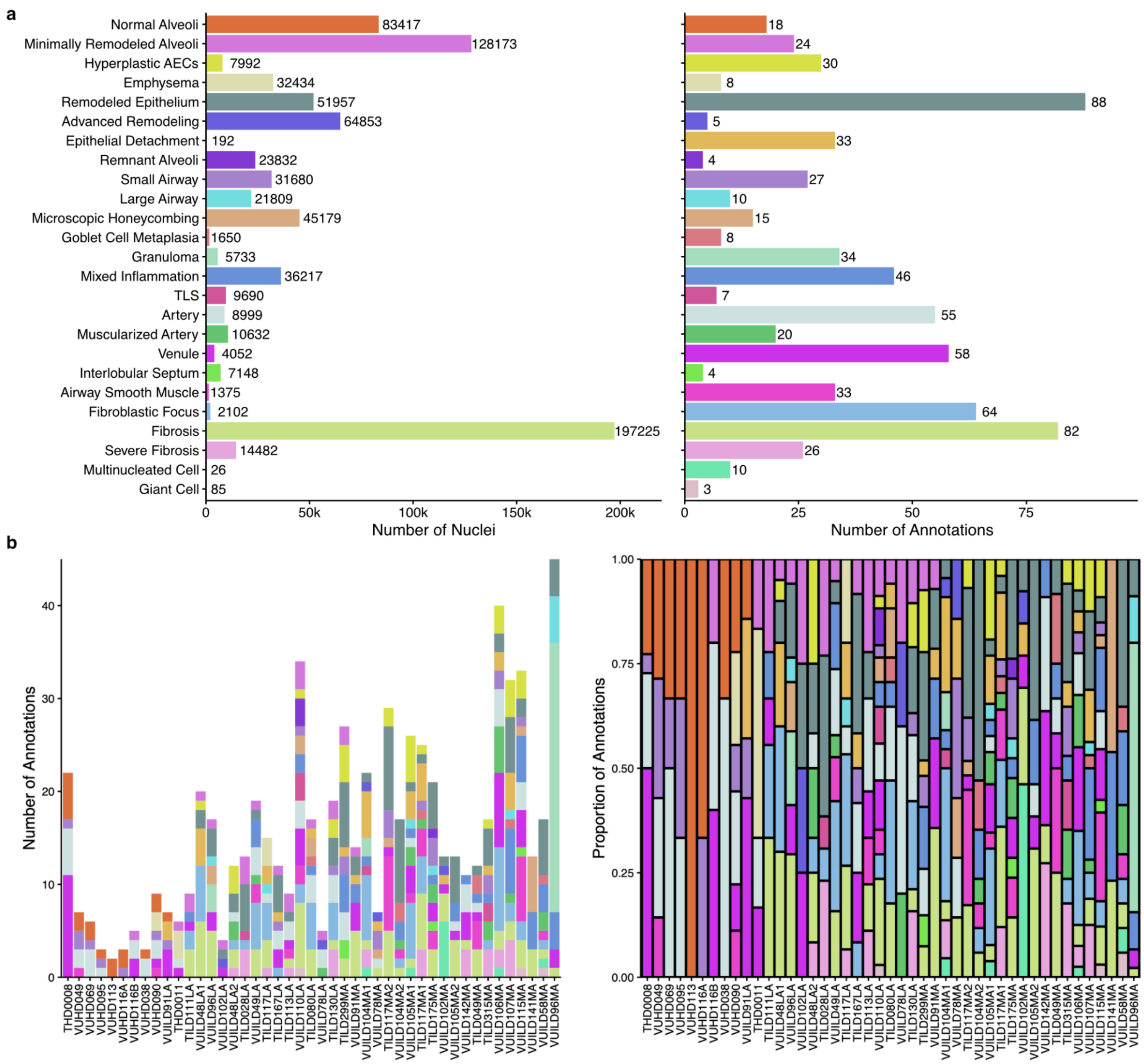

Supplementary Figure 10: Distribution of annotations across samples.
a, The number of nuclei per annotation (left) and total number of annotations of each type (right) across all samples. **b**, Distribution of annotation types across samples, both as counts (left) and as proportions (right) of the total number of annotations for a sample. Samples are organized on the x-axis by increasing percent pathology. Annotations are color-coded the same in both **a** and **b**. Annotation labels include the following abbreviations: AECs = alveolar epithelial cells; TLS = tertiary lymphoid structure.

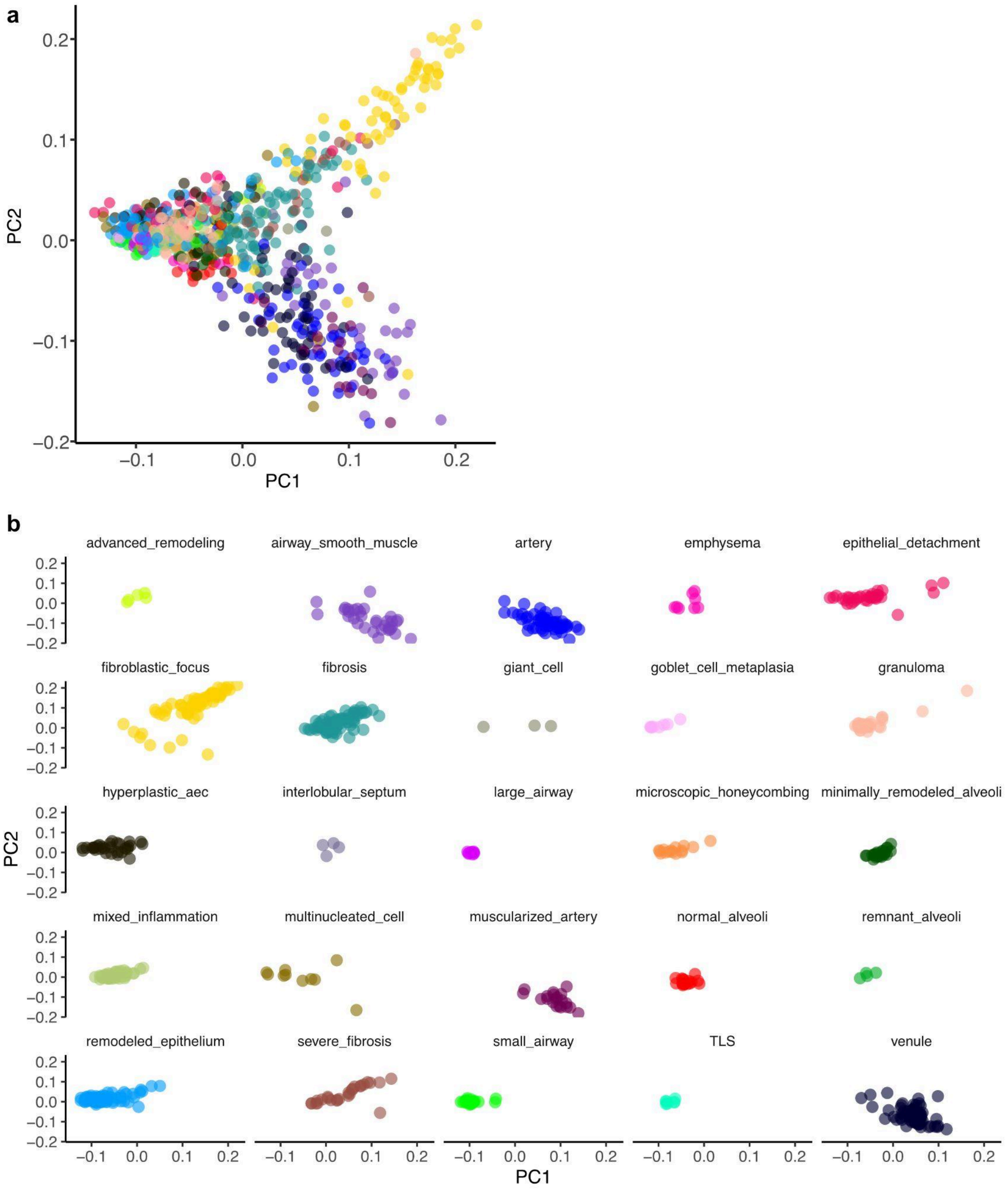

Supplementary Figure 11: PCA of gene expression patterns of annotated histological features.

The gene expression profile of an annotation instance was constructed by aggregating the gene expression of cells assigned to the annotation. Gene expression per annotation instance was normalized by the number of total transcript counts followed by $\log_2$ transformation after adding pseudo-count 1. **a,** PCA plot of histological annotations coloured by annotation type and splitted in 25 panels per annotation type in **b.**

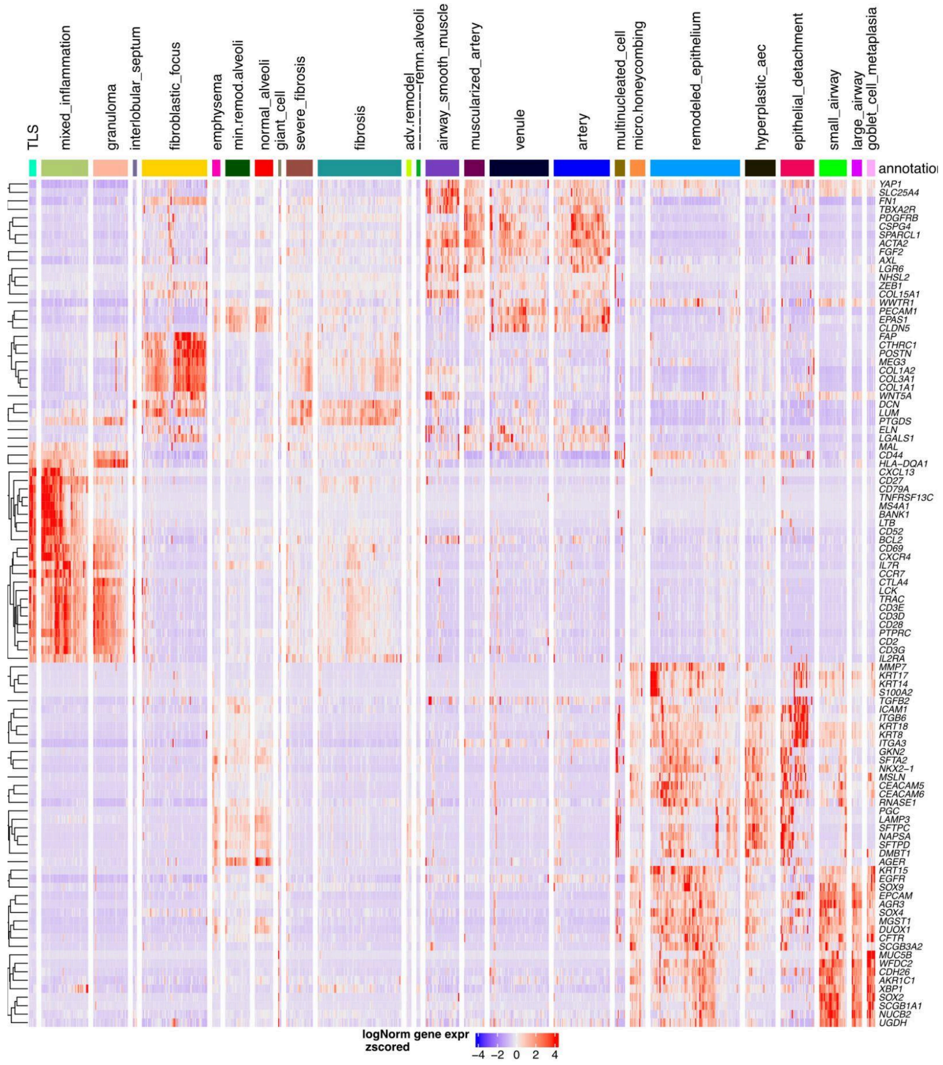

Supplementary Figure 12: Comparison of gene expression patterns underlying all annotated histological features.

Differential gene expression was detected among all annotation types using generalized linear models implemented in *limma*[4] using the $\log_2$CPM transformed gene expression by comparing each annotation to the rest of the annotation groups. The top 100 genes ranked by F-statistics obtained using *topTable* were visualized. Annotation labels include the following abbreviations: adv.remodel = advanced remodeling; min.remod.alveoli = minimally remodeled alveoli; micro.honeycombing = microscopic honeycombing; hyperplastic_aecs = hyperplastic alveolar epithelial cells.

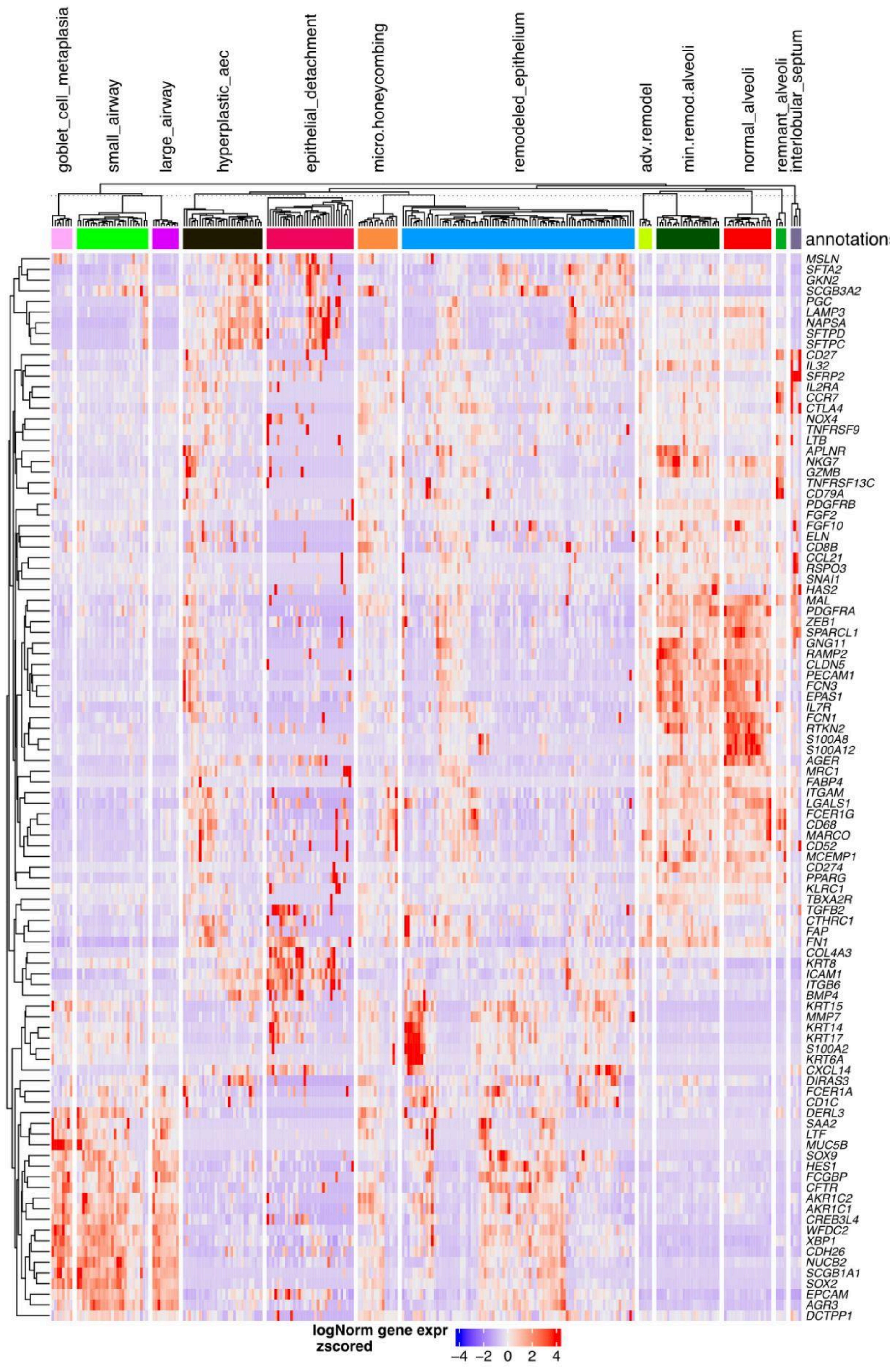

Supplementary Figure 13: Comparison of gene expression patterns underlying epithelial annotations only.

Differential gene expression was detected among epithelial annotations using generalized linear models implemented in *limma*[4] using the log_2CPM transformed gene expression by comparing each annotation to the rest of the annotation groups. The top 100 genes ranked by F-statistics

obtained using *topTable* were visualized. Annotation labels include the following abbreviations: adv.remodel = advanced remodeling; min.remod.alveoli = minimally remodeled alveoli; micro.honeycombing = microscopic honeycombing; hyperplastic_aecs = hyperplastic alveolar epithelial cells.

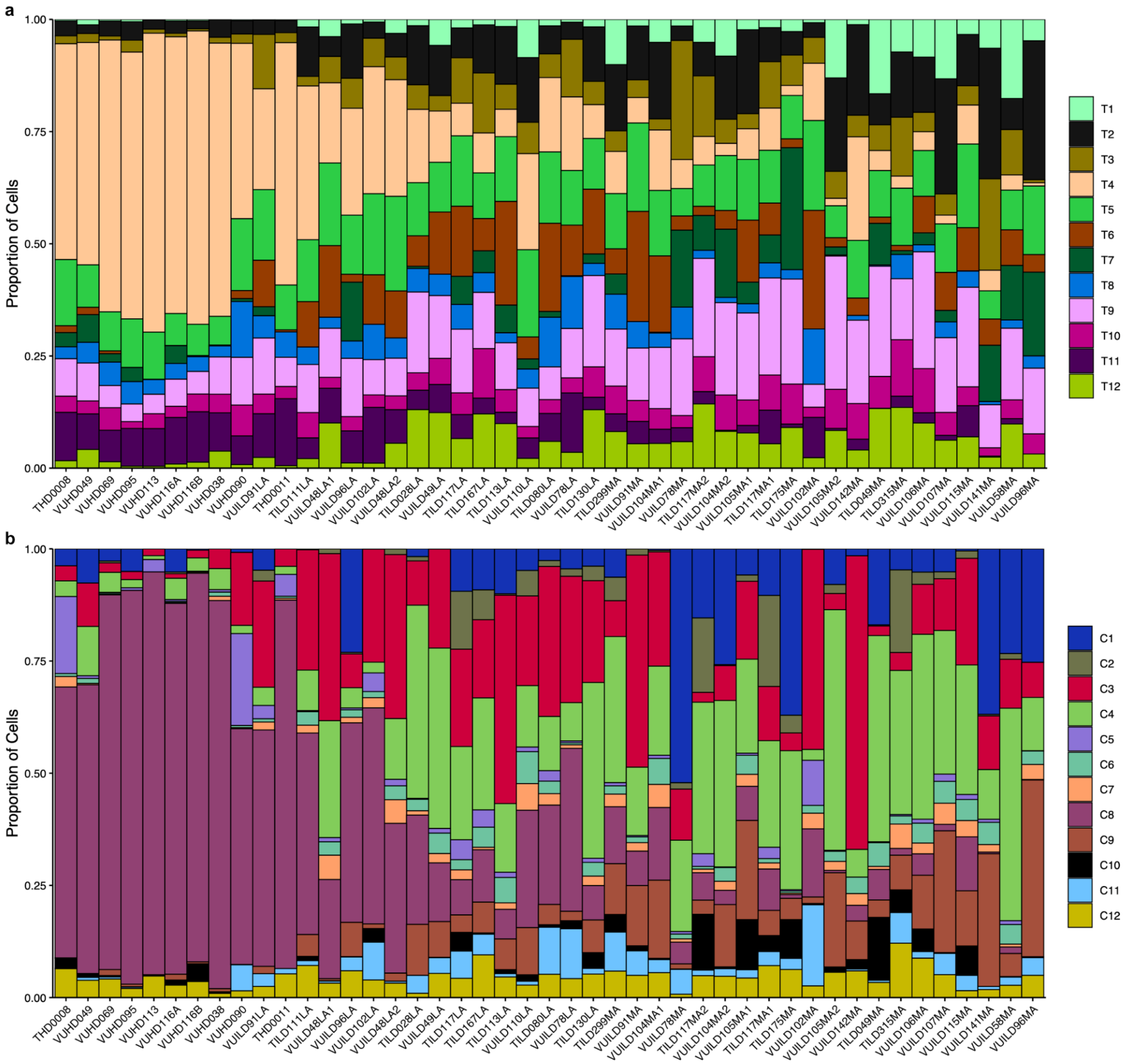

Supplementary Figure 14: Niches assigned to each sample. For each sample, transcripts were assigned to transcript-based niches using GraphSAGE[6] and cells were assigned to cell-based niches using Seurat v5[7]. Bar plots show the proportion of cells assigned to each transcript niche (**a**) and cell niche (**b**) per sample, ordered by percent pathology from lowest to highest. For transcript niches (**a**), niches were determined for each cell by assigning cells to their closest hex bin after hex bin summarization as described in the methods.

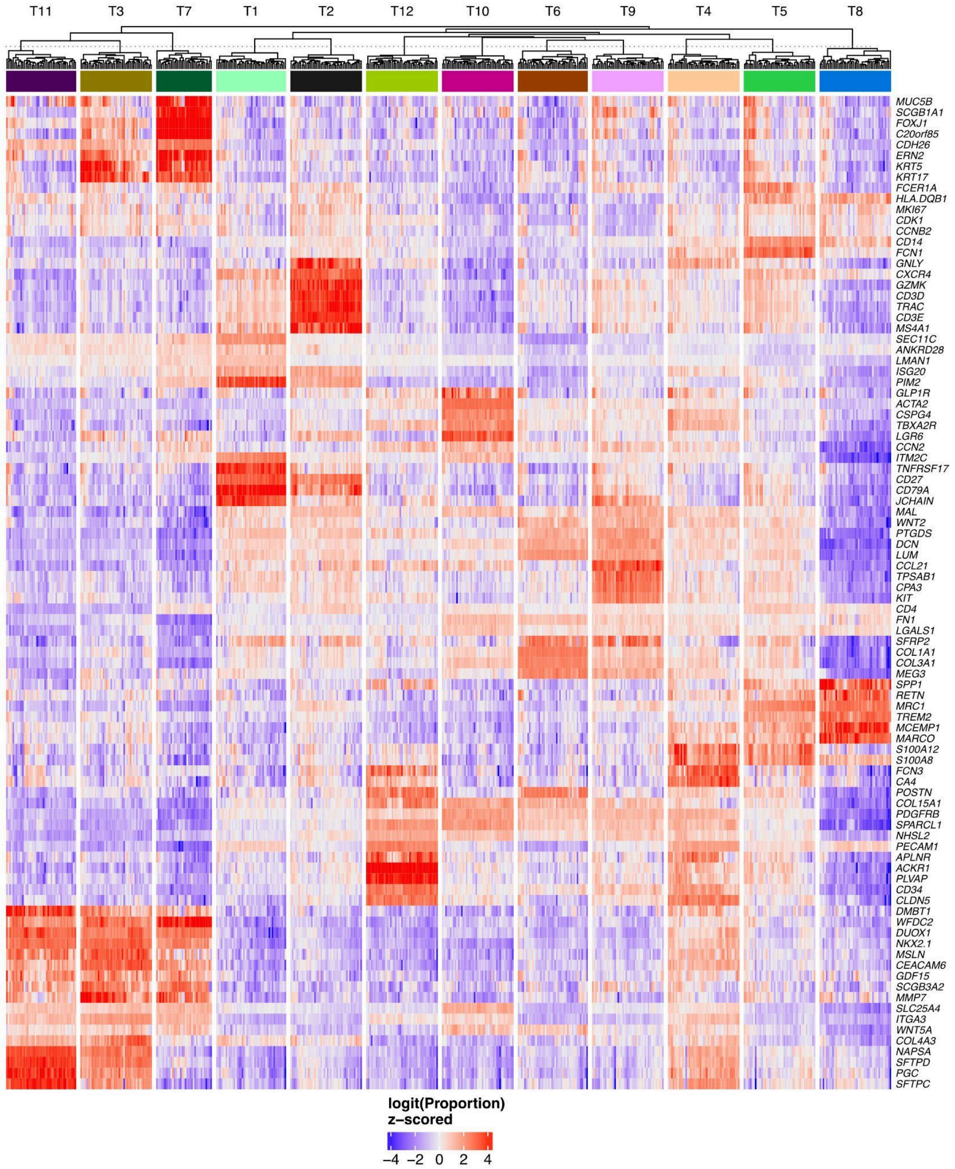

Supplementary Figure 15: Transcript composition of transcript-based niches. Differential proportion testing was performed for finding the representative genes per transcript niche using *propeller*[3] and *limma*[4]. For each transcript niche, gene proportions were calculated per sample, and contrasts were constructed to compare mean proportion in one transcript niche versus the other niches across samples. The top 5 up- and down-regulated genes were selected after ranking by proportion ratio and visualized in heatmaps. Each column in the heatmap shows expressions from an individual sample.

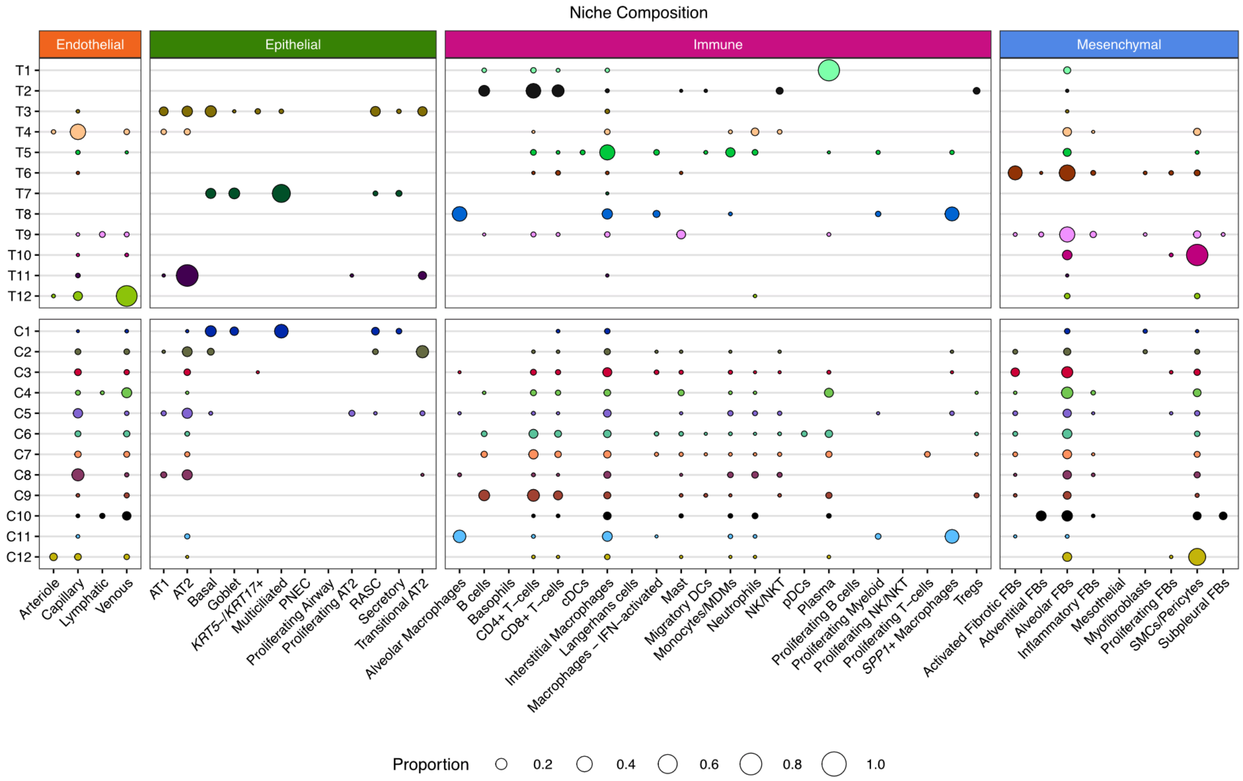

Supplementary Figure 16: cell-type composition of niches.
cell-type composition for transcript- and cell-based-niches (top and bottom, respectively), as a proportion of the total number of cells assigned to the niche (i.e., row sums; rows indicated by gray lines). Proportions under 0.01 are not shown.

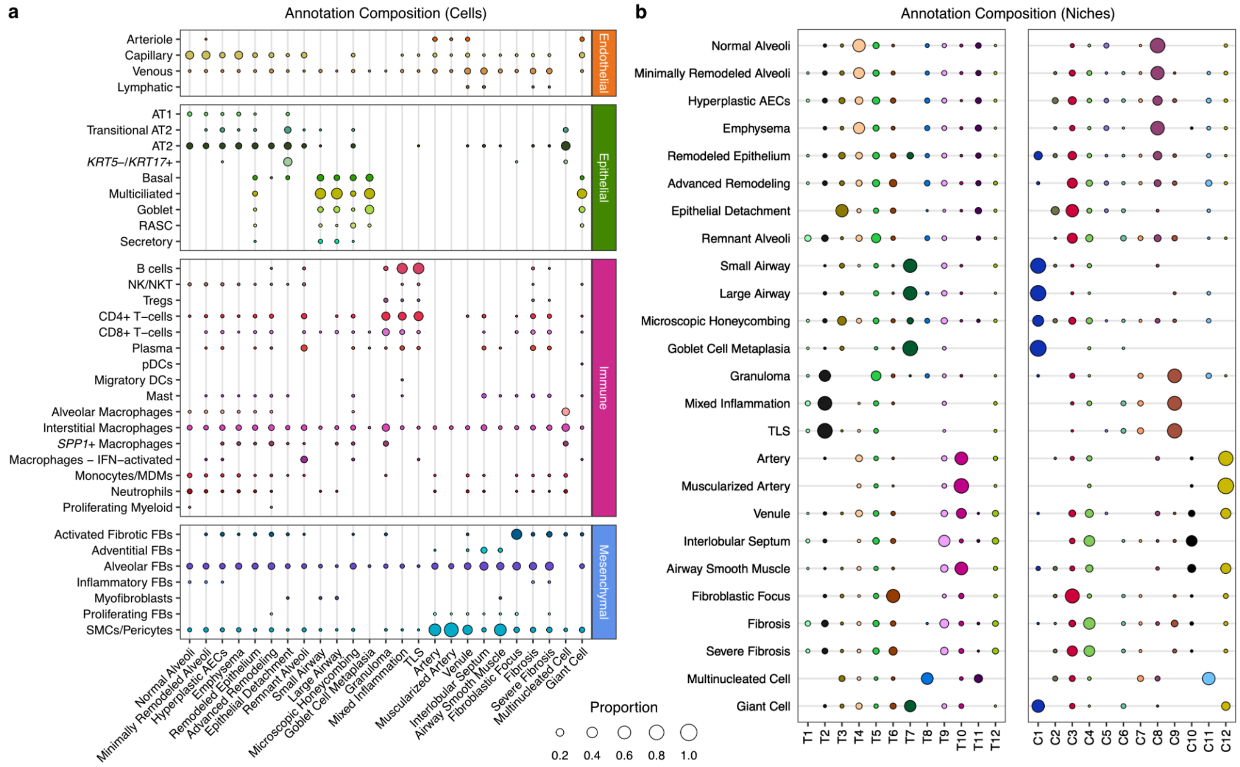

Supplementary Figure 17: cell-types and niches in each annotation.

a, Cell-type composition of each pathologic feature, as a proportion of the number of cells assigned to that annotation (each column sums to 1; columns indicated by gray lines). **b**, The niche composition of all annotations, as a proportion of the number of cells across an annotation (each row sums to 1; rows indicated by gray lines). Panel **b** is the complete version of the plot shown in **Fig. 3d**.

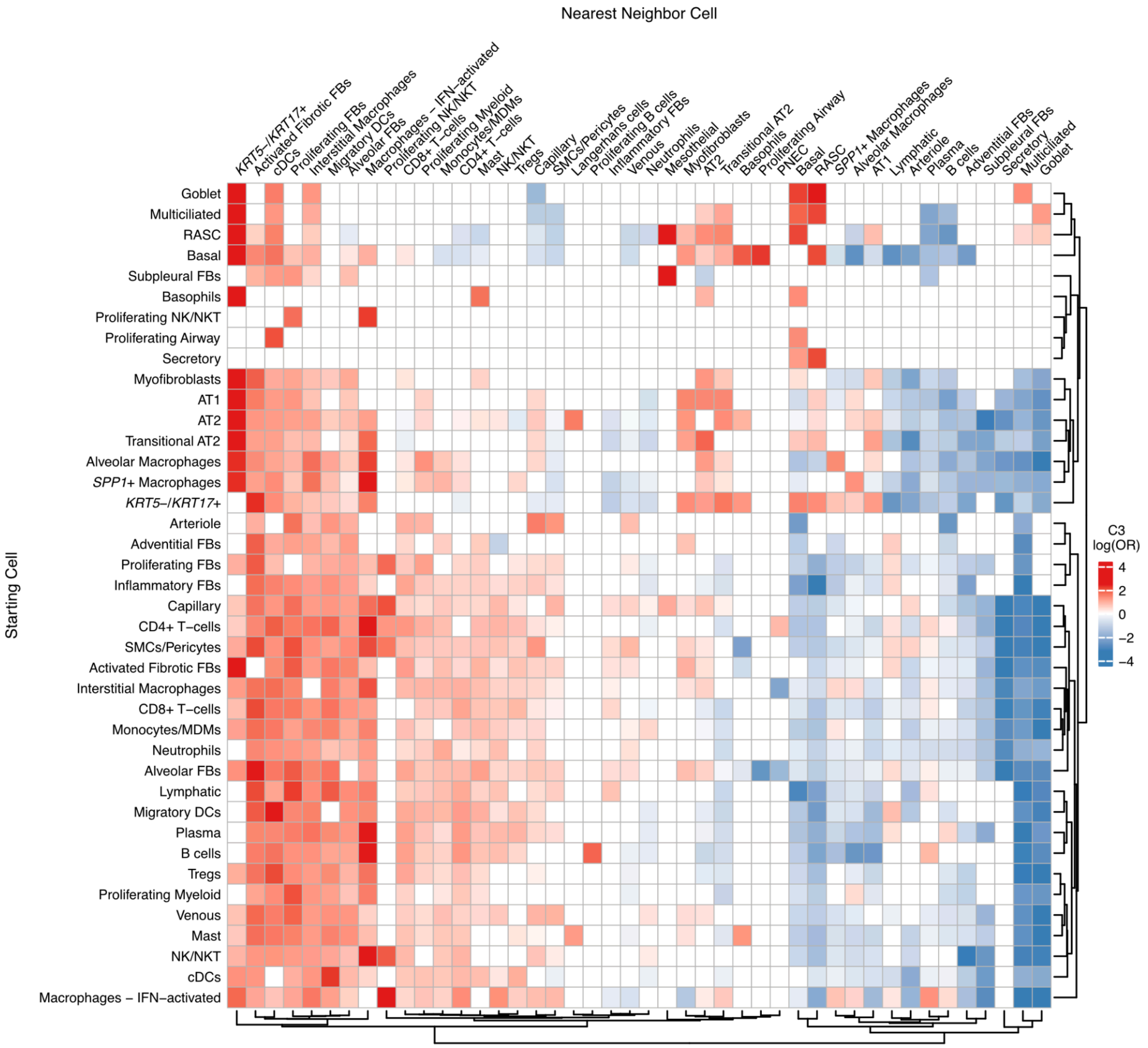

Supplementary Figure 18: C3 niche cell-type proximity results across all samples. Heatmap showing odds ratio indicative of cell-type proximity likelihood within the C3 niche relative to all other cells in the same niche by logistic regression. NAs have been recoded as 0s. See **Supplementary Table 11** for full results for all niches.

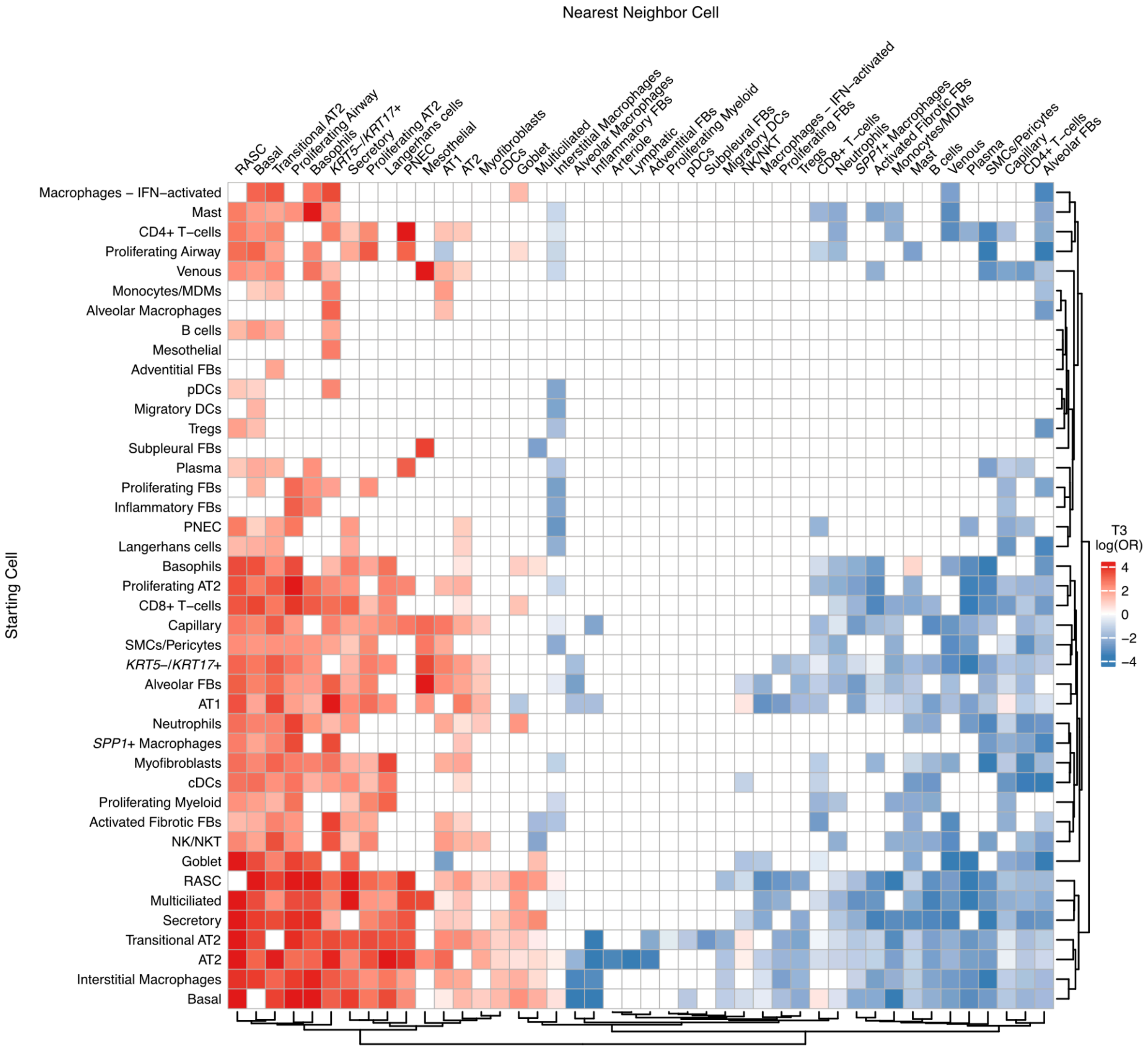

Supplementary Figure 19: T3 niche cell-type proximity results across all samples. Heatmap showing odds ratio indicative of cell-type proximity likelihood within the T3 niche relative to all other cells in the same niche by logistic regression. Positive values represent cell-types that are proximal while negative values represent overall depletion. NAs have been recoded as 0s. See **Supplementary Table 11** for full results for all niches.

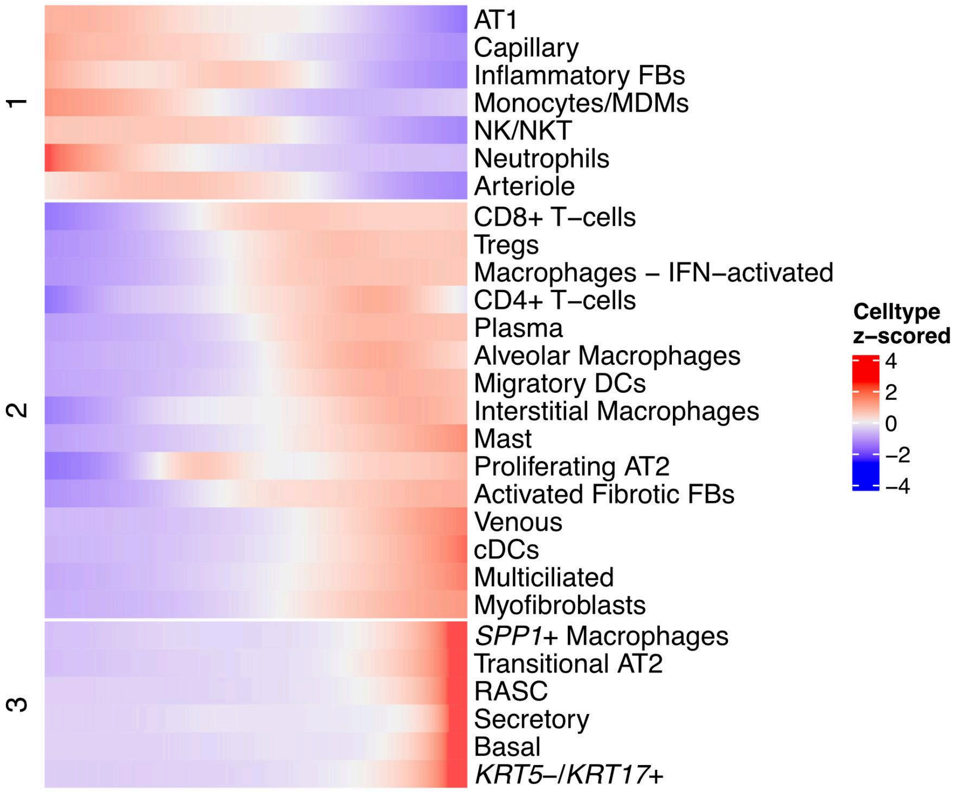

Supplementary Figure 20: Cell-type composition changes along pseudotime.
Cell-type proportion changes in alveolar air spaces along pseudotime. GAM was fitted per cell-type with negative binomial distribution (knots = 12) using cell-type counts across lumens with log of total number of cells per lumen as offset. Cell-types with >=3 count in 10 lumens (36 cell-types) were tested. Association of cell-type abundance with pseudotime was tested using the *associationTest* function in *tradeSeq* with l2fc cutoff 0.5 along 12 time points (nPoints = 12). Cell-types with FDR <= 0.01 were visualized in the heatmap. Cell-types were clustered using spectral clustering from R package *kernlab* and ordered by the peak timepoints.

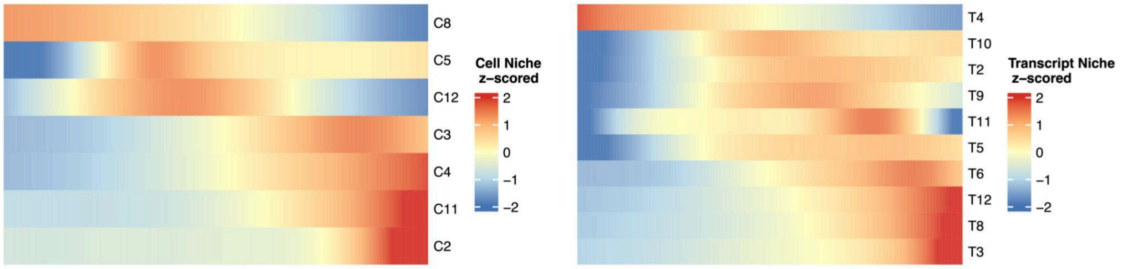

Supplementary Figure 21: Cell and transcript niche composition changes along pseudotime.

Cell niche proportion (**a**) and transcript niche proportion (**b**) changes in alveolar air spaces along pseudotime. GAM was fit per cell-based or transcript-based niche for niches that were found with >= 3 counts in at least 10% of airspaces with negative binomial distribution (knots = 7 for cell-based niches, knots = 10 for transcript-based niches) using niche counts across lumens with log of total cell counts per lumen as offset. Association of niche abundance with pseudotime was tested using the *associationTest* function in *tradeSeq* and nPoints = 7 and 10 for cell niche and transcript niche respectively. Niches with FDR <= 0.01 were visualized in the heatmap.

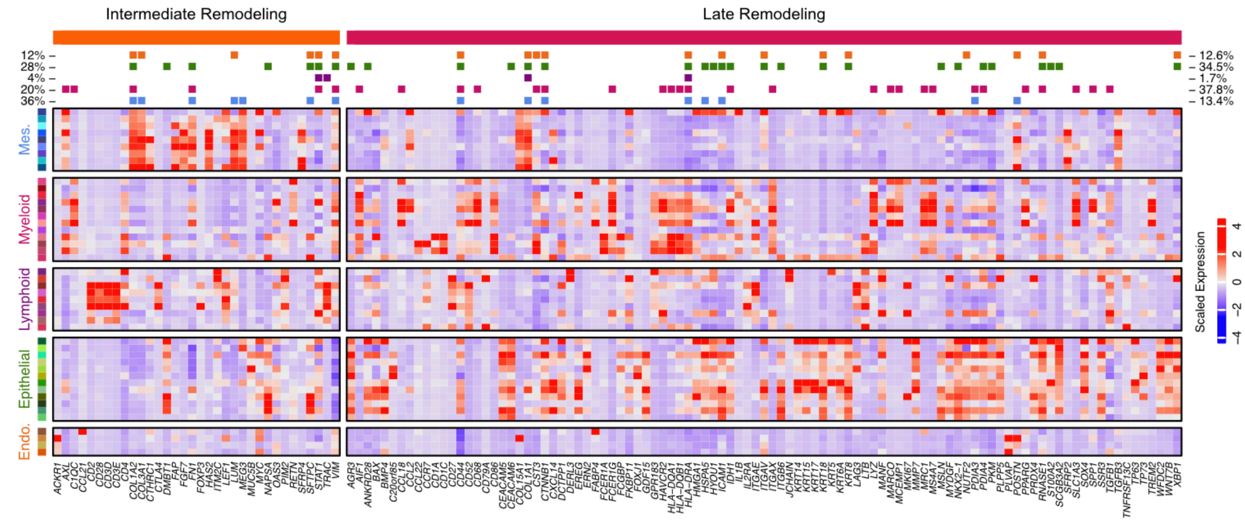

Supplementary Figure 22: Contribution of cell-types to changes in gene expression in intermediate and late remodeling of alveolar airspaces.

Scaled expression across cell-types for the 133 genes with altered expression in the intermediate (34 genes) or late (99 genes) stages of remodeling from the alveolar airspace analysis utilizing only cells that were contained within one of the 1,747 airspaces. Cell-type colors are by lineage as in **Fig. 1**. On the top, boxes are filled in for each gene if it showed a significant change in expression in at least one cell-type across the pseudotime of each of the following lineages: endothelial (orange), epithelial (green), lymphoid (purple), myeloid (pink), and mesenchymal (blue). Percentages were calculated as the number of significant tests (FDR < 0.05) in either the intermediate (orange, left) or late remodeling (red, right) stages that occurred across the pseudotime in all cell-types of each lineage divided by the total number of significant tests for that stage of alveolar remodeling. Endo. = endothelial; Mes. = mesenchymal.

1. Natri, H. M. *et al.* Cell type-specific and disease-associated eQTL in the human lung. *bioRxiv* (2023) doi:10.1101/2023.03.17.533161.
2. Sikkema, L. *et al.* An integrated cell atlas of the lung in health and disease. *Nat. Med.* **29**, 1563–1577 (2023).
3. Phipson, B. *et al.* propeller: testing for differences in cell type proportions in single cell data. *Bioinformatics* **38**, 4720–4726 (2022).
4. Ritchie, M. E. *et al.* limma powers differential expression analyses for RNA-sequencing and microarray studies. *Nucleic Acids Res.* **43**, e47 (2015).
5. Phipson, B., Lee, S., Majewski, I. J., Alexander, W. S. & Smyth, G. K. ROBUST HYPERPARAMETER ESTIMATION PROTECTS AGAINST HYPERVARIABLE GENES AND IMPROVES POWER TO DETECT DIFFERENTIAL EXPRESSION. *Ann. Appl. Stat.* **10**, 946–963 (2016).
6. Hamilton, W. L., Ying, R. & Leskovec, J. Inductive representation learning on large graphs. (2017) doi:10.48550/ARXIV.1706.02216.
7. Hao, Y. *et al.* Dictionary learning for integrative, multimodal, and scalable single-cell analysis. *Nature Biotechnology* **42**, 293–304 (2024).
