# Supplementary material for: Spatial transcriptomics identifies molecular niche dysregulation associated with distal lung remodeling in pulmonary fibrosis
Source: Nat Genet. 2025 Feb 3;57(3):647–58. doi: 10.1038/s41588-025-02080-x (PMC11906353; doi:10.1038/s41588-025-02080-x)
Supplement: Supplementary file 2 — Reporting Summary [file 41588_2025_2080_MOESM2_ESM.pdf]

nature portfolio

Corresponding author(s): Nicholas Banovich
Last updated by author(s): Nov 25, 2024

Reporting Summary

Nature Portfolio wishes to improve the reproducibility of the work that we publish. This form provides structure for consistency and transparency in reporting. For further information on Nature Portfolio policies, see our Editorial Policies and the Editorial Policy Checklist.

Statistics

For all statistical analyses, confirm that the following items are present in the figure legend, table legend, main text, or Methods section.

n/a	Confirmed	
☐	☒	The exact sample size (*n*) for each experimental group/condition, given as a discrete number and unit of measurement
☐	☒	A statement on whether measurements were taken from distinct samples or whether the same sample was measured repeatedly
☐	☒	The statistical test(s) used AND whether they are one- or two-sided<br>*Only common tests should be described solely by name; describe more complex techniques in the Methods section.*
☐	☒	A description of all covariates tested
☐	☒	A description of any assumptions or corrections, such as tests of normality and adjustment for multiple comparisons
☐	☒	A full description of the statistical parameters including central tendency (e.g. means) or other basic estimates (e.g. regression coefficient) AND variation (e.g. standard deviation) or associated estimates of uncertainty (e.g. confidence intervals)
☐	☒	For null hypothesis testing, the test statistic (e.g. *F*, *t*, *r*) with confidence intervals, effect sizes, degrees of freedom and *P* value noted<br>*Give P values as exact values whenever suitable.*
☒	☐	For Bayesian analysis, information on the choice of priors and Markov chain Monte Carlo settings
☐	☒	For hierarchical and complex designs, identification of the appropriate level for tests and full reporting of outcomes
☐	☒	Estimates of effect sizes (e.g. Cohen's *d*, Pearson's *r*), indicating how they were calculated

Our web collection on statistics for biologists contains articles on many of the points above.

Software and code

Policy information about availability of computer code

Data collection	Xenium instrument versions 1.1.2.4 and 2.0.1.0<br>Xenium Analyzer versions xenium-1.1.0.2 and xenium-2.0.0.10<br>Xenium Explorer 3.0.0<br>CytAssist instrument version 2.0.1.6<br>10X Genomics Loupe Browser v8.0.0<br>10X Genomics SpaceRanger 3.0.0
Data analysis	Github code link: https://github.com/Banovich-Lab/Spatial_PF<br><br>ImageJ 2.14.0<br>RStudio Server 2023.06.0+421 "Mountain Hydrangea" Release and RStudio Server 2023.12.1+402 “Ocean Storm” Release<br>R 4.3.0-4 and 4.2.1<br>Python 3.9.7 and 3.12.3<br>Seurat 5.0.1<br>Scanpy v1.8.1<br>RAPIDS v21.8.1<br>RAPIDS cuCIM version 22.12.00<br>CuPy version 11.2.0<br>scikit-image version 3.9.7<br>Alpha shape version 1.3.1<br>QuPath version 0.4.3

GraphSAGE implementation in stellargraph(v1.2.1+python3.8.0)
PyCave (python3.8.4+pycave3.2.1)
propeller 0.99.0
limma 3.50.1
SingleCellExperiment 1.16.0
tradeSeq 1.8.0
kernlab 0.9-32

For manuscripts utilizing custom algorithms or software that are central to the research but not yet described in published literature, software must be made available to editors and reviewers. We strongly encourage code deposition in a community repository (e.g. GitHub). See the Nature Portfolio guidelines for submitting code & software for further information.

Data

Policy information about availability of data

All manuscripts must include a data availability statement. This statement should provide the following information, where applicable:

- Accession codes, unique identifiers, or web links for publicly available datasets
- A description of any restrictions on data availability
- For clinical datasets or third party data, please ensure that the statement adheres to our policy

All data from this study are fully available as supplementary tables or in raw/processed format on GEO: GSE250346. This includes all image data and necessary accompanying files needed to interact with data outputs on the 10X Genomics Explorer software.

Research involving human participants, their data, or biological material

Policy information about studies with human participants or human data. See also policy information about sex, gender (identity/presentation), and sexual orientation and race, ethnicity and racism.

Reporting on sex and gender	Clinically annotated genetic sex was available for 34 of the 35 individuals in the study. Of the reported sex, 14 were female and 20 were male.
Reporting on race, ethnicity, or other socially relevant groupings	Self reported race/ethnicity was available for 34 of the 35 individuals in the study. Of the reported race/ethnicities, 3 reported as African American, 1 as American Indian, and 1 as Hispanic. The remaining subjects reported European ancestry.
Population characteristics	Of the 35 samples, 9 were individuals unaffected by lung disease (declined organ donors). The remaining 26 samples had a form of pulmonary fibrosis including IPF (n = 12), ILD (n = 4), CTD-ILD (n = 2), cHP (n = 4), CPFE (n = 1), NSIP (n = 1), IPAF (n = 1) and Sarcoidosis (n = 1). Ages ranged between 31-64 for controls and 47-70 for pulmonary fibrosis patients. Approximately half (48.6%) of PF patients reported current or prior tobacco use.
Recruitment	Participants were patients of the Clinical Investigator scheduled for a lung transplant surgery. The Clinical Investigator or a member of his research staff approached individuals to discuss the study and invite them to participate.
Ethics oversight	Studies were approved by the local Institutional Review Boards (Vanderbilt IRB nos. 060165 and 171657 and Western IRB no. 20181836)

Note that full information on the approval of the study protocol must also be provided in the manuscript.

Field-specific reporting

Please select the one below that is the best fit for your research. If you are not sure, read the appropriate sections before making your selection.

☒ Life sciences ☐ Behavioural & social sciences ☐ Ecological, evolutionary & environmental sciences

For a reference copy of the document with all sections, see nature.com/documents/nr-reporting-summary-flat.pdf

Life sciences study design

All studies must disclose on these points even when the disclosure is negative.

Sample size	The final sample size was 45 samples from 35 donors, consisting of 9 unaffected controls and 26 samples with pulmonary fibrosis. Sample size calculations were not performed. Sample size was chosen based on sample availability and the number of samples feasible to run together in the same Xenium runs. While this dataset contains a relatively small number of individuals, it is the largest imaging-based spatial transcriptomic study of the human lung reported to date and was sufficient to generate a large dataset containing more than 1.6 million cells.
Data exclusions	Cells with a large size, low number of counts, or unexpectedly high numbers of counts were filtered out based on current best practices.
Replication	Key findings from Xenium data were replicated with orthogonal technology - Visium HD.
Randomization	Data generation was randomized with respect to disease and control samples.

Blinding

This study was unblinded. Blinding would not be possible because major differences in histology are readily observable both between control and disease and between different disease diagnoses.

Reporting for specific materials, systems and methods

We require information from authors about some types of materials, experimental systems and methods used in many studies. Here, indicate whether each material, system or method listed is relevant to your study. If you are not sure if a list item applies to your research, read the appropriate section before selecting a response.

Materials & experimental systems

n/a	Involved in the study
☒	☐ Antibodies
☒	☐ Eukaryotic cell lines
☒	☐ Palaeontology and archaeology
☒	☐ Animals and other organisms
☒	☐ Clinical data
☒	☐ Dual use research of concern
☒	☐ Plants

Methods

n/a	Involved in the study
☒	☐ ChIP-seq
☒	☐ Flow cytometry
☒	☐ MRI-based neuroimaging

Plants

Seed stocks

Report on the source of all seed stocks or other plant material used. If applicable, state the seed stock centre and catalogue number. If plant specimens were collected from the field, describe the collection location, date and sampling procedures.

Novel plant genotypes

Describe the methods by which all novel plant genotypes were produced. This includes those generated by transgenic approaches, gene editing, chemical/radiation-based mutagenesis and hybridization. For transgenic lines, describe the transformation method, the number of independent lines analyzed and the generation upon which experiments were performed. For gene-edited lines, describe the editor used, the endogenous sequence targeted for editing, the targeting guide RNA sequence (if applicable) and how the editor was applied.

Authentication

Describe any authentication procedures for each seed stock used or novel genotype generated. Describe any experiments used to assess the effect of a mutation and, where applicable, how potential secondary effects (e.g. second site T-DNA insertions, mosiacism, off-target gene editing) were examined.
